# Respecting the Old and Loving the Young: Emoji-Based Sarcasm Interpretation Between Younger and Older Adults

**DOI:** 10.3389/fpsyg.2022.897153

**Published:** 2022-05-19

**Authors:** Jing Cui

**Affiliations:** College of Foreign Languages and Cultures, Xiamen University, Xiamen, China

**Keywords:** sarcasm, smiling emoji, ambiguity, age, relationship

## Abstract

The interpretation of sarcasm relies on many cues and constraints. In computer-mediated communication (CMC), paralinguistic cues, such as emoticons and emoji, play an important role in signaling sarcastic intention. Smiling emoji have been claimed to be a marker of sarcasm among Chinese senders. Shared knowledge between the sender and the recipient, such as age and relationship, has a substantial effect on irony or sarcasm interpretation. However, hardly any research has been done to integrate the two factors to explore their joint effect on sarcasm interpretation. The present study investigated the interaction effect of these factors on the interpretation of ambiguous statements accompanied by a smiling emoji. Two experiments were conducted to investigate the differences between younger and older adults in making judgments about ambiguous statements accompanied by a smiling emoji. The results showed that sender age and sender–receiver relationship have disparate influences on younger and older adults’ interpretation of emoji-based ambiguous statements. For younger adults, sender age and sender–receiver relationship were significantly associated with the perceived sarcasm of emoji-based ambiguous statements, whereas for older adults, sender age had a null effect on the sarcastic interpretation of emoji-based ambiguous statements, but relationship was an important cue that might impact their interpretation.

## Introduction

In social interaction, people hold different attitudes toward people of different “orders of relationship”^1^ (Chang and Holt, 1991, p. 253). This communication pattern in China underscores the social relationships stemming from the doctrines of Confucianism. An ordering and sequencing principle—“precedence of the old over the young”—emphasizes a communication pattern that older people should be loving and friendly and the younger should be pious and respectful. In other words, age and relationship are influential factors in organizing human relationships (Chang and Holt, 1991; Yan and Sorenson, 2004). There are a variety of forms to express elder respect, including linguistic respect for seniors in interactions (Sung and Kim, 2003; Sung and Dunkle, 2009).

Sarcasm, a specific form of irony, targets an individual or a proposition with a critical or negative attitude (Kreuz and Glucksberg, 1989). The speaker of sarcasm is perceived as saying something and meaning something like the opposite (Grice, 1975). Such an inherently ambiguous nature sometimes makes it difficult to decide whether the utterance is sarcastic (Davidov et al., 2010; Chaudhari and Chandankhede, 2017). According to the constraint satisfaction model, in the process of irony interpretation, multiple cues to ironic intent are processed rapidly and in parallel with the statement itself (Katz, 2005; Pexman, 2008). Shared knowledge, beliefs, and experiences between the speaker and the addressee are expected to facilitate the interpretation process (Gibbs, 1986; Clark, 1996; Shamay-Tsoory et al., 2005). Common knowledge and information, such as speaker age and speaker–addressee relationships, are clues that might affect the identification of the speaker’s intention. Therefore, whether the communication pattern of “respecting the old and loving the young” would adapt to sarcasm interpretation or not is worth studying.

Speakers of sarcasm usually intend their addressees to glean their intended meaning. There are indications that a statement may still be interpreted sarcastically in the absence of any social or contextual cues. A diverse range of communicative strategies is used to convey their intentions. Paralinguistic evidence, such as a specific facial expression and a distinctive prosody, are cues in favor of successful irony or sarcasm interpretation (e.g., Rockwell, 2000; Attardo et al., 2003; Bryant, 2010). However, computer-mediated communication (CMC) might be ambiguous in interpretation because of the scarcity of contextual and non-verbal cues abundantly available in face-to-face (FtF) communication (Kiesler et al., 1984; O’Neill, 2010). Based on Walther’s (1996) hyperpersonal model, this ambiguity could be overcome, because senders are able to selectively display themselves, and receivers will rely on minimal cues and idealize perceptions of senders. Emoticons and emoji are common paralinguistic cues in CMC developed to supplement text communication and shape perceptions (Hancock, 2004; Jiang et al., 2018; Weiß et al., 2020).

“Emoticons” are sideways typographical marks that originated in symbols from the American Standard Code for Information Interchange (ASCII), and “emoji” are Japanese-specific Unicode characters that gained particular popularity in Asia (e.g., Herring and Dainas, 2017; Prada et al., 2018). Previous studies have found cultural variabilities in people’s use of emoticons/emoji to indicate ironic or sarcastic intentions. People in Western cultures are more inclined to express ironic intentions explicitly and directly by means of the overtly expressed wink (;)) and tongue face (: P) (Derks et al., 2008; Filik et al., 2016; Thompson and Filik, 2016). In contrast, people in Chinese culture tend to express ironic intentions implicitly and indirectly through a smiling face (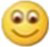) that is explicitly polite and happy, but implicitly sarcastic (Zhou et al., 2017; de Seta, 2018). Smiling emoji is a WeChat^2^-specific emoji prevalent among the Chinese. This type of proprietary emoji is pictorial in appearance and differs from the Unicode one registered on Emojipedia (Ge and Herring, 2018; de Seta, 2018). It has been suggested that age and relationship could also affect the use of the smiling emoji. Among older adults, the smiling emoji is a genuine smile representing happiness and hospitality, while younger generations assign a sarcastic meaning to the emoji, and seldom use the smiling emoji sarcastically when communicating with their family members (Zhou et al., 2017).

The present study attempts to investigate how the principle of “respecting the old and loving the young” affects people’s sarcasm interpretation, and whether sender age and sender–receiver relationship have interaction effects on emoji-based sarcasm interpretation in Chinese culture.

### The Effect of Relationship on Irony Usage and Interpretation

The idea that the speaker–addressee relationship appears to have an effect on irony use is documented in the literature. For instance, closeness is reported to increase the likelihood of using irony. Kreuz (1996) found that close relationships were positively correlated with the use of sarcasm. This indicates that individuals in close relationships are more inclined to use sarcasm than people in distant relationships. It has been suggested that people in friendly relationships frequently use irony, accounting for about 8% percent of their conversational content (Gibbs, 2000). Individuals in solidary relationships (i.e., close, liking, and familiar) were also reported to use irony more frequently than speakers in non-solidary relationships (Pexman and Zvaigzne, 2004). In addition, the likelihood of using sarcasm varies with the change in situations. Channon et al. (2005) demonstrated that it is proper to use sarcasm with a family member, but less appropriate with people in a formal situation, such as with an interviewer in a job interview. However, a close relationship is not equally effective for irony interpretation. Kreuz et al. (1999) demonstrated that the manipulation of closeness exerts an influence on the appropriateness of a statement rather than on irony ratings. Pexman and Zvaigzne (2004) found that relationship had no significant effect on rating the politeness of irony. They suggested that solidary relationships did not modulate irony perception, which was supposed to be an all-or-none type of phenomenon, rather than one graded by degrees (Kreuz et al., 1999). Predictably, if a sarcastic/non-sarcastic dichotomy is employed in evaluating an individual’s sincerity, the effect of a close relationship on irony interpretation would be noticeable.

To better understand the effect of relationships on the use and interpretation of irony, some scholars have attempted to manipulate relationships with some other factors. Relationships can affect the use of irony when interactions involve direct statements (Pexman et al., 2010). It has been suggested that people in close relationships care little about face-saving, so that direct irony is preferable, whereas indirect irony is more face-saving and tends to be used for individuals in distant relationships (Brown and Levinson, 1978). Slugoski and Turnbull (1988) distinguished relationship from relationship affect, suggesting the inappropriateness of subsuming or evaluating the former under the latter factor. Relationship should not be confounded with relationship affect because relationship can be judged from liking, but the same cannot be said for relationship affect. Therefore, a relationship affect factor—liking—was added to their analysis model. Their results suggested that liking was much more effective in irony interpretation than relationships. A liking relationship between the speaker and the addressee facilitates the perception of literal insults as ironic compliments, whereas a disliking relationship increases the possibility of interpreting literal compliments as ironic insults. Relationship fails to reveal any effect on the interpretation of literal insults, but it impacts participants’ interpretation of literal compliments on the condition that the speaker and the addressee like each other.

As suggested, relationships play a vital role in the use of irony, but they are less important for the interpretation of irony. However, because of these contradictory results, many other factors might constrain the effect of relationships on irony interpretation. The manipulation of relationships with other factors appears to be essential for irony or sarcasm interpretation.

### The Effect of Relationship on Emoticon/Emoji Usage

The lack of paralinguistic cues as in FtF communication can sometimes challenge individuals’ disclosure of feelings and emotions in CMC, thus hindering the process of advancing to a more intimate relationship. Emoticons/emoji help to convey affective information to individuals, which in turn can shape the sender–receiver relationship in CMC (Miller et al., 2016; Gesselman et al., 2019). The frequency and the functions of using emoticons/emoji vary with different relationships. As mentioned, people in friendly relationships are more apt to contact each other *via* text-based communication, and they exhibit a higher preference for using emoticons/emoji in their conversations (Derks et al., 2008). Emoticon/emoji usage is associated with friendship development. Riordan et al. (2014) demonstrated that friends are more likely to use emoticons than strangers in instant message exchanges. The more emoticons are used, the more probable individuals are to develop closer relationships with others (Utz, 2000). In sarcasm-oriented studies, researchers have found that individuals in friendly relationships have a high frequency of using emoticons (more than 72 types of emoticons) to indicate their sarcastic intentions (Thompson and Filik, 2016), and emoticons are important devices for indicating sarcastic emotions in emails between friends (Whalen et al., 2009). Therefore, it is predicted that individuals in close relationships will have a propensity to use emoticons/emoji.

### Age Differences in Irony Use and Interpretation

Evidence to determine whether there are age differences in the use of irony or sarcasm is scarce. Howman and Filik (2020) measured the use of irony for both younger and older adults by means of the sarcasm self-report scale, and they found that younger adults were more inclined to use sarcasm than older adults. In contrast to the idea that elderly people rarely use irony or sarcasm, Liptak et al. (2014) reported that affectionate sarcasm was the most frequently used humor type employed by older adults with mild cognitive impairment and their caregivers to relieve tension and create a friendly social environment. However, the participants (i.e., both older adults and caregivers) in the study were 60 years or older, so age differences were not reflected by contrasting with younger groups. There was also a pre-existing relationship between older adults and caregivers in that situation. That is, the conversation was between people of “lower status” (i.e., older adults) and individuals of “higher status” (i.e., caregivers), where older adults were reported to be more inclined to produce sarcastic humor than caregivers in order to equalize themselves at the same level (Saunders et al., 2011).

Age also contributes to the current findings in irony comprehension. Phillips et al. (2015) found age-related differences in irony interpretation using non-literal criticism (i.e., sarcasm) between younger, middle-aged, and older adults. Compared with younger and middle-aged adults, elderly people were reported to have no difference in interpreting literal exchanges, but were more inclined to interpret non-literal utterances literally rather than sarcastically. Such age deficits in sarcasm interpretation have also been demonstrated by Pomareda et al. (2019). In their study, older adults were found to have a higher likelihood to interpret non-literal language literally, to rate non-literal criticism kinder, and to rate non-literal compliments meaner than younger adults. Likewise, Rothermich et al. (2021) investigated the effect of age on irony processing using video-based naturalistic tasks. Their results showed that younger and middle-aged adults were more capable of comprehending sarcasm and teasing than older adults. Additionally, older adults were inclined to interpret sarcasm as friendlier than younger and middle-aged adults. Overall, it is expected that the age of the sender is influential on irony use and that the age of the participants is a crucial factor for irony interpretation.

### Age Differences in Emoticon/Emoji Usage and Interpretation

User experiences with Internet tools and digital products, such as smartphones, influence their understanding and command of products (Kim and Yang, 2016; Zhou and Gui, 2017). There are variations in the capacity, frequency, and motivations for using Internet technology between people born with digital gadgets and those born without digital products (Huffaker and Calvert, 2005; Vodanovich et al., 2010). Thus, user age is negatively associated with the use of new media and technology (Rosen et al., 2013; Forgays et al., 2014; Hauk et al., 2018). These age differences were also found in the usage and understanding of emoticons and emoji. Young people have a higher frequency of using emoticons/emoji (Danet and Herring, 2007; An et al., 2018; Herring and Dainas, 2020; Weiß et al., 2020), and they favor a wider range of emoji to express their emotions and feelings (An et al., 2018; Weiß et al., 2020). Young groups endow some emoji with more sophisticated and conventional functions, while older people regard emoji as a reinforcement of the verbal message (Herring and Dainas, 2020).

Young and old users also differ in their interpretations of emoticons/emoji. In a study of emoticon recognition, Hsiao and Hsieh (2014) found significant differences between younger and older adults. Their research revealed that older adults not only assessed emoticons more positively and pleasantly but also displayed more positive responses than younger adults. Likewise, Weiß et al. (2020) found an ascent in the perceived positivity of the smiley with increasing age. Older adults feel more pleasure and positive emotions toward emoticons and emoji. Zhou et al. (2017) also found age differences in the interpretation of the smiling emoji. Young users assigned a sarcastic meaning to the emoji, but old people interpreted the emoji literally as an indication of happiness. The age of 30 is reported to be a dividing line for assigning emoji with disparate meanings (Herring and Dainas, 2020). Users over 30 tend to interpret emoji literally. They have difficulty in identifying the motivations and intentions defined by people less than 30 years old. An et al. (2018), for instance, found that users aged 16–25 years defined the smiling emoji as negative or neutral in valence, but people over 36 years old used the emoji to convey positive sentiments.

Such age differences in emoticon/emoji interpretation might be due to a positivity bias. According to the socioemotional selectivity theory (SST), the perception of time left in life would influence an individual’s socioemotional goals (Carstensen et al., 1999; Carstensen, 2006). As age increases, people show a preference for more agreeable experiences and more positive emotions in social relationships. Older people tend to hold a more positive attitude in their lives and prefer more positive information (Mather and Carstensen, 2005). Therefore, older adults have a higher tendency to endow emoticons/emoji with more positive meanings and emotions.

To our knowledge, however, there is no study concerned about individuals’ perception of emoji based on sender age. As is suggested in the above-mentioned literature, people of different ages would use and interpret emoji differently. Hence, whether sender age would affect receivers’ interpretation of the smiling emoji is worth noting.

### Age Differences in the Use and Interpretation of Non-verbal Cues That Indicate Ironic Intention

A wide range of paralinguistic cues has been used to indicate ironic intentions in both FtF communication and CMC. However, the recognition of these cues changes with increasing age. Happé et al. (1998) found age-related improvements in identifying social cues because healthy elderly people have a better state of theory of mind (ToM) ability than young adults. However, Orbelo et al. (2005) reported age-related deficits in prosody identification among elderly people regarding the interpretation of non-literal statements resulting from declining right hemisphere function. Henry et al. (2013) also demonstrated that because of their ToM descents, older adults had more difficulty interpreting the intentions and beliefs in senders’ facial expressions and gestures. Elderly people were also reported to have a declined ability to identify facial expressions, prosody, and gestures, which are effective cues for sarcasm and teasing interpretation, because their working memory, inhibition, and abstract did not fare as well as that of younger people (Rothermich et al., 2021). In addition, age deficits were found in emotion perception and perspective-taking, so that elderly people had age-related declines in recognizing cues and constraints that help to successfully interpret sarcastic statements (Phillips et al., 2015).

In CMC, the wink emoticon is an indicator of ironic intention (e.g., Filik et al., 2016; Thompson and Filik, 2016). The wink emoticon is reported to facilitate young adults’ irony interpretation in an eye-tracking experiment conducted by Howman and Filik (2020). In contrast, older adults have a lower probability of recognizing the effect of the wink emoticon in interpreting irony. In Chinese culture, the smiling emoji has been taken as a genuine smile to indicate happiness and hospitality by older adults, whereas the innovative meaning of the smiling emoji—acting as a mysterious smile to express sarcasm and speechlessness^3^—is more pervasive among young adults (Zhou et al., 2017). Based on age differences in the interpretation of irony and the smiling emoji, it is expected that older adults are less likely to interpret emoji-based ambiguous statements sarcastically than younger adults.

### The Present Study

Pexman and Zvaigzne’s (2004) findings demonstrated that a solidary relationship has no influence on irony ratings, because irony is not graded by degrees, it is rather an all-or-none concept (Kreuz et al., 1999). Therefore, the present study employed a sarcastic/non-sarcastic dichotomy to determine whether the effect of a close relationship would be highlighted in evaluating senders’ sincerity. As suggested earlier, 30 years old might be the age of endowing emoji with diverse meanings and functions. Therefore, we employed a sample composed of younger adults less than 30 years old and older adults over the age of 40. Apart from the age of participants, sender age and sender–receiver relationship were also manipulated in the scenario.

In Experiment 1, sender age and sender–receiver relationship have already been set beforehand. Building on the idea that people might have different criteria for judging the age of others and the relationship with other people based on their personal experiences, Experiment 2 makes supplements by delegating powers to participants themselves in making judgments of sender age and sender–receiver relationship, allowing them to set up their exclusive background relationship context. This operation also helps to make sender age and sender–receiver relationship salient.

We attempted to address the following two research questions:

1. How does sender age and sender–receiver relationship influence the interpretation of emoji-based ambiguous statements of younger and older adults, respectively?
2. Is there any interaction effect between sender age and sender–receiver relationship on the sarcastic interpretation of emoji-based ambiguous statements?

## Experiment 1

### Methods

#### Materials and Design

Twenty-four scenarios were devised. Each scenario consisted of a situation that included two characters (i.e., the sender and the receiver). One neutral context sentence briefly introduced the sender–receiver relationship (close or distant) and sender age (young or old), followed by the sender delivering a statement to the receiver. The age of the sender was shown by means of two age prefixes (i.e., “*Xiao*” and “*Lao*”), and honorifics to indicate age differences were defined as “老 + 姓” (i.e., “*Lao* [old] *+* surname”) and “小 + 姓” (i.e., “*Xiao* [young/little] + surname”). The relationship was indicated by expressions like “best friend” and “colleague,” to express the closeness between the sender and the receiver (see Table 1).

**Table 1:** Example materials in Experiment 1.

*1. Target item*
小李和老张是忘年交。周末，小李带老张去露营。老张:你选了个好地方 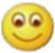
Q1. 老张觉得露营地好吗? ( ) A. 好 B. 不好
Q2. 老张的话的友好程度? 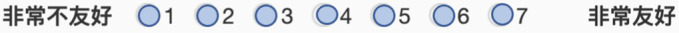
Xiao Li invited his best old friend Lao Zhang to go camping last Sunday Lao Zhang: The place you chose was quite good 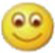
Q1. How did Lao Zhang think of the camping site? A. Good B. Bad
Q2. How friendly do you think Lao Zhang’s comment is? 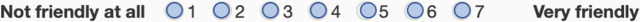
*2. Filler item* 老张和小王是同事。他们约定一块早起去登山。第二天早上，老张:你起得太晚了 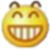
Q1. 老张是不是不开心? ( ) A.是 B.否
Q2. 老张的话的友好程度? 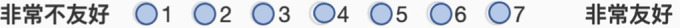
Lao Zhang and Xiao Wang are colleagues. They agreed to get up early and go hiking together. The next morning, Lao Zhang: You get up too late 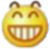
Q1. Was Lao Zhang unhappy? A. Yes B. No
Q2. How friendly do you think Lao Zhang’s comment is? 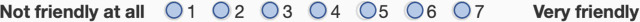

The target statements were 12 superficially positive utterances accompanied by a smiling emoji. Such positive verbal messages could be interpreted as either literal praise or sarcastic criticism, depending on the context. Another 12 statements were filler items, consisting of 10 superficially negative statements and two superficially positive statements. These 12 statements were followed either by a grinning face (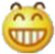) or a shocking face (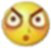). All the items were mixed up, so that participants could not guess that the real target of the experiment was the smiling emoji. All three factors were within-subjects. Therefore, this experiment was composed of a 2 (relationship: close vs. distant) ^*^ 2 (sender age: young vs. old senders) ^*^ 2 (literality: sarcastic vs. non-sarcastic) design.

Each scenario had two questions. The first question asked participants to decide sender belief (e.g., “How did Lao Zhang think of the camping site?”—*Good/Bad*). The second question was a 7-point rating scale about sender intention (e.g., “How friendly do you think Lao Zhang’s comment is?” 1 = *Not friendly at all*, 7 = *Very friendly*). Sender belief tapped into the understanding of the sincerity of the statement. Sender intention tapped into the sender’s attitude toward the other character in the context. The remaining 12 filler items were followed by comprehension questions, but the questions were not associated with the interpretation of the target statement (see Table 1 for an example). Together with a question to test the purpose of the investigation (“What is the main purpose of the present study?”), each list consisted of a total of 25 items. Before the experiment, a pilot study was conducted to select the items.

#### Pilot Study

To objectively select the items in the formal experiment, 19 participants (*M* = 24.95, SD = 6.79, 10 females) were asked to rate the positivity of the statements used in the materials *via* an online questionnaire. None of them would participate in the subsequent formal experiment. Participants were asked to rate 80 potential statements (i.e., 40 superficially positive and 40 superficially negative statements) by using multiple-choice questions (“Is the sentence positive or negative?”). The statements were presented without any context nor any emoji. Positive statements that were interpreted positively were recorded as PP (positive text–positive response), while those interpreted as negative were recorded as PN (positive text–negative response). Negative statements with negative interpretation were recorded as NN (negative text–negative response), and negative statements with positive interpretation were recorded as NP (negative text–positive response). A t-test was used to compare the responses to different statements. There was a significant difference between PP and PN, *t* (18) = 64.12, *p* < 0.001. Negative statements were significantly likely to be interpreted as negative in meaning by participants, *t*(18) = 64.12, *p* < 0.001. However, there was no significant difference between PP and NN interpretation [*t*(18) = 0.29, *p* = 0.78], indicating that the interpretation of the positively valenced statements was not significantly different from that of the negatively valenced statements. We selected 14 positively valenced and 10 negatively valenced statements with the highest scores.

#### Participants

A total of 175 native Mandarin speakers (109 females, accounting for 62.3%) participated in this experiment. All reported using smartphones, the WeChat app, and emoji in their daily conversations. Of these, 87 were younger participants recruited from Xiamen University (their ages ranged from 18 to 30 years old, *M_young_* = 21.86, SD = 2.81; *N_female_* = 57, accounting for 65.5%). The remaining 88 were older participants recruited by snowball sampling (their ages ranged from 40 to 58 years old, *M_old_* = 45.38, *SD* = 4.66, *N_female_* = 52, accounting for 59.1%). We firstly found several volunteers aged between 40 and 59 years old, and asked them to refer us to other potential subjects who might be willing to participate in this experiment. The participants continued a chain-referral process until we obtained enough sample population. Ethical approval was obtained by the College of Foreign Languages and Cultures in Xiamen University, China.

#### Procedure

Participants were tested face-to-face in small groups of up to 15 people at a time in a lab setting. All participants received a booklet containing a consent form, an instruction, and the basic demographic information about their age, gender, and their use of smartphones, WeChat, and emoji. This was followed by the experimental task. The scenarios in the rating task involved 4 conditions resulting from the interaction of age and relationship (i.e., *Close-Young, Distant-Young, Close-Old, and Distant-Old*; see Table 2). Hence, the 25 items were distributed at random into four separate lists. Each participant was randomly assigned a list. All participants gave informed consent and signed the consent forms, and they received 5 RMB in reward for participation. The task lasted about 15 min.

**Table 2:** Example scenarios in Experiment 1.

*Close-Young*
小李和小刘是好朋友。周末，小李带小刘去露营。小刘:你选了个好地方。
Xiao Li invited his best friend Xiao Liu to go camping last Sunday. Xiao Liu: The place you chose was quite good
*Close-Old*
老张和小李是忘年交，周末他们一起去露营。老张:你选了个好地方。
Lao Zhang and Xiao Li are best old friends. They went camping last Sunday. Lao Zhang: The place you chose was quite good
*Distant-Young*
周末,小王剬司组织去露营，小王负责安排整个活动。新同事小李:你选了个好地方。
Last Sunday, the company of Xiao Wang organized a camping trip. Xiao Wang was responsible for this activity. New colleague Xiao Li: The place you chose was quite good
*Distant-Old*
周末，小王剬司组织去露营，小王负责安排整个活动。董事长老马:你选了个好地方。
Last Sunday, the company of Xiao Wang organized a camping trip. Xiao Wang was responsible for this activity. The chairman of the company Lao Ma: The place you chose was quite good

## Results and Discussion

### The Literality of Emoji-Based Ambiguous Statements

**Figure 1:**
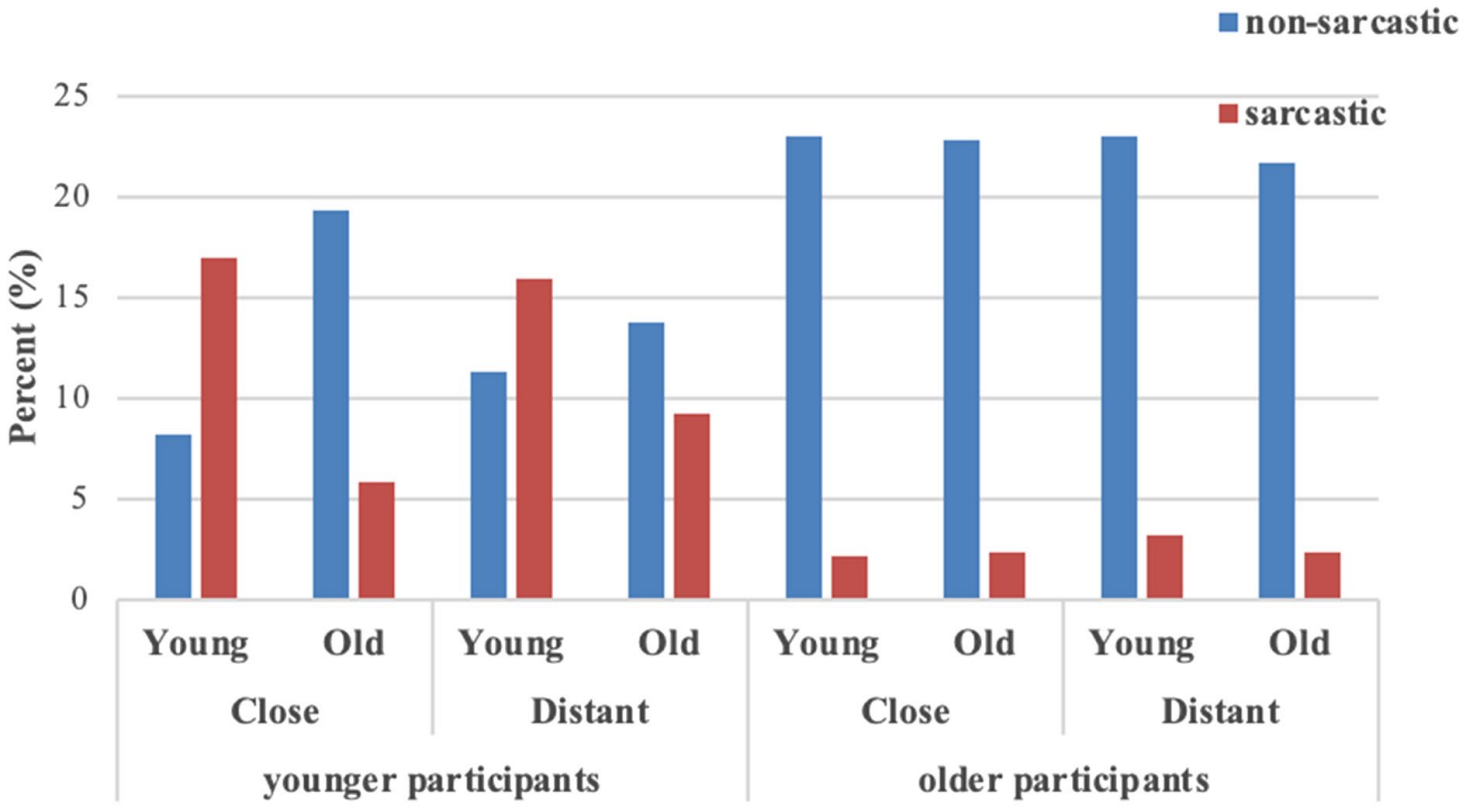
Frequencies of sarcasm in Experiment 1.

We carried out the analysis using log-linear models in SPSS 25 to measure whether there was any interaction among participant age (younger or older), relationship (close or distant), sender age (young or old), and literality (sarcastic or non-sarcastic) in emoji-based sarcasm interpretation. The collected data (*N* = 2,100) was more than five times the cells (*n* = 16) in a multiway table, which satisfied the criterion of the log-linear model (Tabachnick and Fidell, 2007). Figure 1 displays the relative percentages of each condition.

The results of the goodness-of-fit test showed that the probability (*p* = 0.25) was greater than 0.05, indicating that there was no significant statistical difference between the unsaturated model and the saturated model with all interactive options. That is, this model fully reflects the relationship between the four variables. The probability value of likelihood ratio chi-square test showed that the fourth-order and above effects were significant ($\chi_{\left(1\right)}^{2}$ = 9.83, *p* < 0.01).

To break down this effect, separate chi-square tests on sender age, relationship, and literality were performed separately for younger and older participants. For younger participants, there was a significant association among sender age, relationship, and literality, $\chi_{\left(1\right)}^{2}$ = 20.08, *p* < 0.001; but this was not true in older participants, $\chi_{\left(1\right)}^{2}$ = 0.73, *p* = 0.39. This indicated that for younger participants, sender age and sender–receiver relationship were two factors that might affect their emoji-based sarcasm interpretation. In contrast, neither sender age nor relationship exerted influence on older participants’ interpretation of emoji-based statements.

Separate chi-square tests on relationship and literality were performed by sender age in the data of younger participants. The results revealed that for young senders, relationship had a marginally significant effect on literality, $\chi_{\left(1\right)}^{2}$ = 4.48, *p* = 0.04; this was true in old senders, $\chi_{\left(1\right)}^{2}$ = 17.15, *p* < 0.001. Odds ratios indicated that the odds of young senders’ being perceived as sarcastic in close relationships were 1.46 higher than in distant relationships, but the odds were only 0.44 for old senders. This revealed that, among younger participants, young senders in close relationships were more likely to be perceived as sarcastic than young senders in distant relationships when delivering emoji-based ambiguous statements. In contrast, old senders in close relationships were 2.27 times lower to be perceived as sarcastic than old senders in distant relationships. Therefore, the analysis seems to reveal a fundamental difference between young and old senders: young senders in close relationships have a slightly higher possibility to be perceived as sarcastic than those in distant relationships, whereas old senders in distant relationships are more likely to be perceived as sarcastic than those in close relationships.

We split the data of younger participants by the relationship variable. Separate chi-square tests on sender age and literality were performed. The results revealed that in close relationships, sender age and literality interacted significantly, $\chi_{\left(1\right)}^{2}$ = 104.07, *p* < 0.001; this was true in distant relationships, $\chi_{\left(1\right)}^{2}$ = 17.72, *p* < 0.001. Odds ratios indicated that the odds of young senders in close relationships being perceived as sarcastic were 6.94 higher than the odds of old senders in close relationships. In comparison, young senders in distant relationships had 2.11 higher odds of being perceived as sarcastic than old senders in distant relationships. Thus, the analysis reveals that in both close and distant relationships, young senders are more likely to be perceived as sarcastic than old senders of emoji-based ambiguous statements.

### Rating of Friendliness

The second question asked participants to rate, on a 7-point scale, their interpretation of the sender’s attitude toward the recipient of an ambiguous statement accompanied by a smiling emoji. It consisted of a 2 (sender age: young vs. old) ^*^ 2 (relationship: close vs. distant) research design, with sender age and relationship as the within-subjects factors.

According to the hypothesis, the effect of sender age and sender–receiver relationship on the judgment of sender attitude were analyzed separately for younger and older participants. The mean score and standard deviation of younger and older participants’ judgment of the friendliness based on sender age and relationship are presented in Tables 3 and 4, respectively.

**Table 3:** The mean score and standard deviation of friendliness by younger participants.

		*Sender age*	
		Young	Old
*Relationship*	Close	*M* = 3.59, SD = 1.64	*M* = 4.95, SD = 1.57
	Distant	*M* = 3.38, SD = 1.72	*M* = 4.17, SD = 1.73

**Table 4:** The mean score and standard deviation of friendliness by older participants.

		*Sender age*	
		Young	Old
*Relationship*	Close	*M* = 5.67, SD = 1.40	*M* = 5.69, SD = 1.39
	Distant	*M* = 5.44, SD = 1.45	*M* = 5.65, SD = 1.35

The results of younger participants’ interpretation showed that there was a significant interaction between relationship and sender age in judging the friendliness of the sender’s statement, *F*(1, 260) = 7.30, *p* = 0.007, $\eta_{p}^{2}$ = 0.03. Simple effect tests showed that young senders in close relationships were rated as significantly less friendly than old senders in close relationships (MD = −1.36, *p* < 0.001); old senders in distant relationships were perceived to be significantly friendlier than young senders in distant relationships (MD = 0.79, *p* < 0.001); young senders in close relationships were rated more but not significantly friendlier than young senders in distant relationships (MD = 0.21 *p* = 0.16); and old senders in close relationships were perceived significantly friendlier than old senders in distant relationships (MD = 0.78, *p* < 0.001).

The results of older participants’ interpretation showed that there was no significant interaction between relationship and sender age in the effect of the friendliness of the sender’s statement, *F*(1, 263) = 1.65, *p* = 0.20, $\eta_{p}^{2}$ = 0.006. This indicated that for older participants, sender age and relationship were not significantly associated with the friendliness of emoji-based ambiguous statements. The remaining main effects were also non-significant (*ps* > 0.05).

To summarize the results of Experiment 1, sender age and sender–receiver relationship have interaction effects on younger adults’ perception of emoji-based ambiguous statements, but this is not true in older adults. When delivering an ambiguous statement accompanied by a smiling emoji, young senders were more likely to be perceived as sarcastic than old senders in both close and distant relationships. Young senders in close relationships had a slightly higher tendency to express sarcastic meanings than those in distant relationships. However, old senders in distant relationships were significantly more likely to express sarcastic meanings than those in close relationships. Conversely, neither sender age nor sender–receiver relationship had any significant effect on older adults’ perception of emoji-based ambiguous statements.

Likewise, for the perception of speaker intention, sender age and sender–receiver relationship have an interaction effect on younger adults’ perception of the friendliness of the statement. However, this is not true for older adults. For younger adults, when delivering ambiguous statements accompanied by a smiling emoji, old senders were perceived as friendlier in both close and distant relationships than young senders, and older senders in close relationships were friendlier than those in distant relationships. However, young senders were perceived as friendly in both close and distant relationships. In contrast, for older participants, sender age or sender–receiver relationship exerted no significant difference in judging the friendliness of emoji-based ambiguous statements.

## Experiment 2

Experiment 1 focused on the interpretation of the combination of the ambiguous text and the smiling emoji. Little attention was paid to the effect of the emoji. It is worth noting whether sender age and sender–receiver relationship have any effect on the understanding of the emoji, and how these differences are related to the interpretation of ambiguous statements. In addition, Experiment 1 set sender age as well as sender–receiver relationship beforehand. The age of the sender was shown by two age prefixes—“*Lao*” and “*Xiao.*” However, there is a subversion to normalcy in the usage of these honorifics, especially among young people. For instance, young people would call their peers “老 + 姓” (i.e., “*Lao +* surname”) to indicate their intimacy (Hung, 1993). The already-defined age of the sender designed in Experiment 1 might exclude the novel use of the two honorifics, especially in close relationships. Additionally, the standard by which the sender–receiver relationship is judged appears to vary from person to person. Therefore, sender age and sender–receiver relationship should be judged by the participants themselves based on the context and their own experiences.

In Experiment 2, the participants were asked to judge the age of the sender and the relationship between the characters. We added questions about the judgment of sender age and sender–receiver relationship, and we also set a free response question about the meaning of the smiling emoji in the context. In this way, sender age, sender–receiver relationship, and the emoji could be made more salient by being mentioned repeatedly in the questions. Therefore, we did not employ filler items in this experiment.

### Methods

#### Materials and Design

This experiment involves 4 scenarios selected from Experiment 1. These four positively valenced statements were accompanied by a smiling emoji which could be interpreted either as literal praise or sarcastic criticism, depending on the context. All three factors were within-subjects. Therefore, this experiment was composed of a 2 (relationship: close vs. distant) ^*^ 2 (sender age: young vs. old) ^*^ 2 (literality: sarcastic vs. non-sarcastic) design.

Following each scenario were five questions. The first three were multiple-choice questions asking participants to judge sender age (e.g., “When you read the context, what age did you set for Lao Zhang?”—*Young/Old*), the sender–receiver relationship (e.g., “When you read the context, what was the relationship you have set for the two characters?”—*Close/Distant*), and sender belief (e.g., “How did Lao Zhang think of the camping site?”—*Good/Bad*). The fourth was a free response question asking the subjects to state “What did the smiling emoji mean in the context?” The fifth question was a 7-point rating scale about sender intention (e.g., “How friendly do you think Lao Zhang’s comment is?” 1 = *Not friendly at all*, 7 = *Very friendly*). The first two questions tapped into the idea that different people might have different ideas or judgments about the age and the relationship of the characters. The free response question of the emoji meaning tapped into participants’ perception of the sender’s intent. Since we repeatedly reminded participants about sender age and the sender–receiver relationship as well as the emoji, the purpose of the study was disclosed to the participants. Hence, we diminished the filler items in this task, and directly targeted ambiguous statements accompanied by a smiling emoji.

#### Participants

A new sample of 344 native Mandarin speakers (213 females, accounting for 61.9%) took part in this experiment, including 191 young participants (their ages ranged from 18 to 30 years old, *M_young_* = 21.41, SD = 2.91; *N_female_* = 116, accounting for 60.7%) recruited from Xiamen University, and 153 older participants (their ages ranged from 40 to 59 years old, *M_old_* = 50.35, SD = 5.15, *N_female_* = 97, accounting for 63.4%) recruited by snowball sampling. Several volunteers aged between 40 and 59 years participated in our experiment at first, and then they continued a chain-referral process until we obtained enough sample population. All participants were reported to use smartphones, WeChat, and emoji in their daily lives. This experiment was conducted with ethical approval from the College of Foreign Languages and Cultures in Xiamen University, China.

#### Procedure

Following the same procedure as in Experiment 1, the 4 items ^*^ 4 conditions were distributed into four separate lists with each condition appearing once in each list, drawing on the basis from a 4^*^4 latin square. After providing a written consent, each participant was randomly assigned with a list. A group of 15–20 participants were tested at a time face-to-face in a lab setting. The task lasted about 10 min. Each participant got 5 RMB in reward for their participation.

## Results and Discussion

### The Literality of Emoji-Based Ambiguous Statements

**Figure 2:**
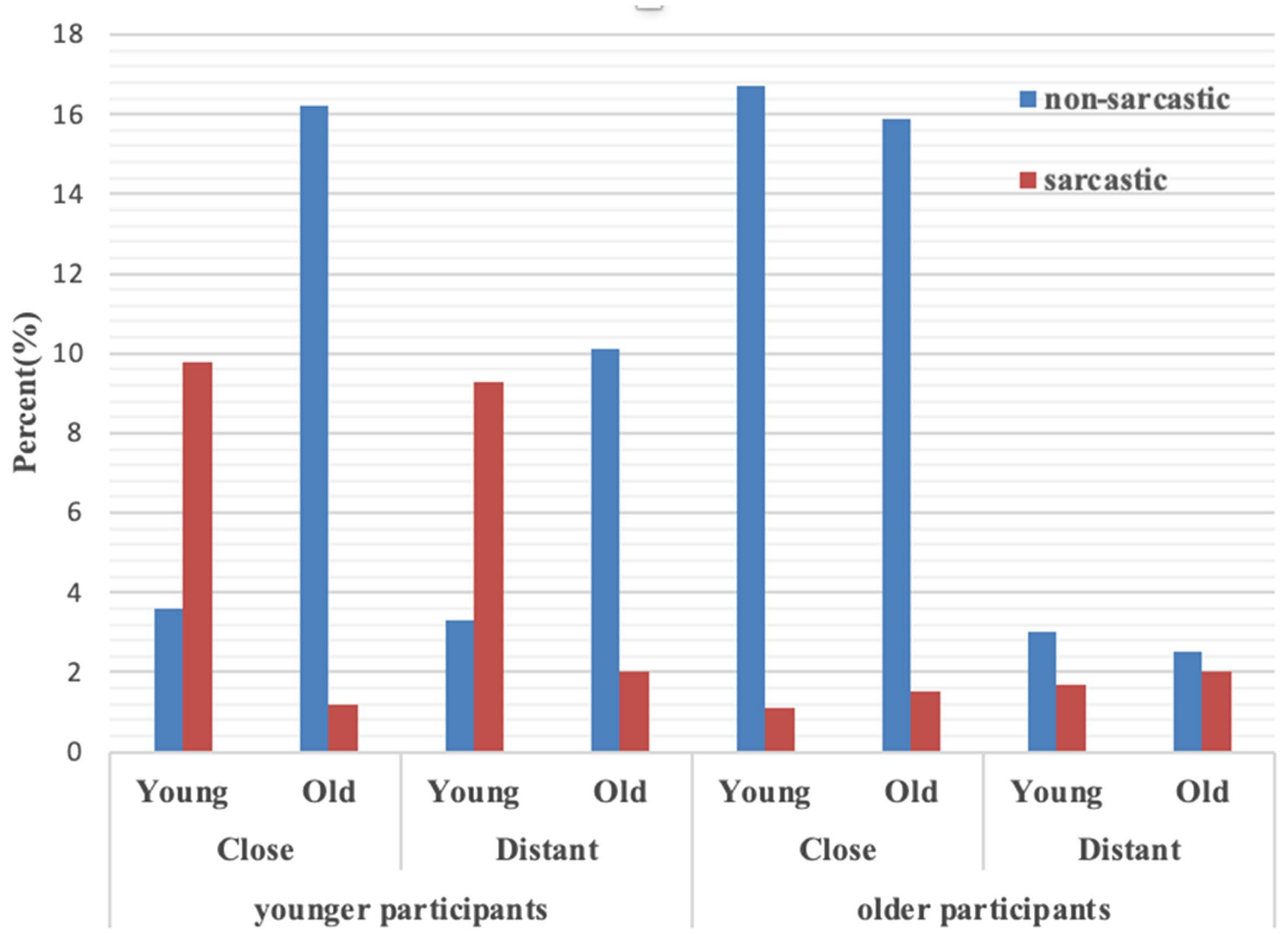
Frequencies of sarcasm in Experiment 2.

As in Experiment 1, an exploratory multiway frequency analysis was conducted to construct a log-linear model in SPSS 25. The collected data (*N* = 1,376) were more than five times the number of cells (*n* = 16) in the multiway table, satisfying the criterion of the log-linear model. The relative percentages of each condition are displayed in Figure 2.

The results of the goodness-of-fit test showed that the probability (*p* = 0.25) was greater than 0.05, indicating that the saturated model fully reflected the relationship between the four variables. The results of the probability value of the likelihood ratio chi-square test showed that the four-way interaction (*k* = 4) was non-significant ($\chi_{\left(1\right)}^{2}$ = 2.83, *p* = 0.09). However, the test of all effects of the third-order and above ($\chi_{\left(5\right)}^{2}$ = 211.03, *p* < 0.001), and the second-order and above ($\chi_{\left(11\right)}^{2}$ = 665.71, *p* < 0.001), were significant.

The results of the partial association tests showed that 10 of the 14 effects were significant. There was a significant association among participant age, relationship, and literality, $\chi_{\left(1\right)}^{2}$ = 26.16, *p* < 0.001, indicating that participants of varying ages interpreted emoji-based statements differently based on the sender–receiver relationship. A significant association among participant age, sender age, and literality was found, $\chi_{\left(1\right)}^{2}$ = 115.87, *p* < 0.001, revealing that participants perceived emoji-based statements differently depending on sender age. Participant age was also significantly associated with sender age ($\chi_{\left(1\right)}^{2}$ = 29.88, *p* < 0.001) and relationship ($\chi_{\left(1\right)}^{2}$ = 48.30, *p* < 0.001), indicating that younger and older participants judged sender age and the sender–receiver relationship in diverse ways. Sender age and relationship were significantly associated with literality of the statements, *ps* < 0.001. There were main effects for participant age, relationship, and literality, *ps* < 0.05. The remaining third-order interactions and the main effect of sender age were non-significant, *ps* > 0.05.

We broke down the 4-way interaction and performed a chi-square test on relationship, sender age, and literality separately for younger and older participants. For younger participants, there was a significant association among sender age, relationship, and literality, $\chi_{\left(1\right)}^{2}$ = 5.84, *p* = 0.02; but this was not true for older participants, $\chi_{\left(1\right)}^{2}$ = 0.05, *p* = 0.83. This indicates that sender age and the sender–receiver relationship are two factors that would affect younger participants’ interpretation of emoji-based ambiguous statements, but the two factors exert no influence on older participants’ interpretation of emoji-based statements.

Separate chi-square tests on relationship and literality were performed by sender age in the data of younger participants. The results revealed that relationship did not significantly interact with literality in young senders, $\chi_{\left(1\right)}^{2}$ = 0.05, *p* = 0.83; however, it was significant in old senders, $\chi_{\left(1\right)}^{2}$ = 10.32, *p* = 0.001. Odds ratios indicated that for young senders, the odds of being perceived sarcastically in close and distant relationships were 0.95, but only 0.36 in old senders (i.e., the odds of old senders in distant relationships being perceived as sarcastic were 2.81 times higher than those in close relationships). This reveals a fundamental difference between young and old senders: the perception of young senders’ emoji-based statements is not associated with their relationship, while old senders are more likely to be perceived as sarcastic in distant relationships than in close relationships.

Likewise, we split the data by the relationship variable. Separate chi-square tests on sender age and literality were performed based on close and distant relationships. The results revealed that in close relationships, sender age significantly interacted with literality, $\chi_{\left(1\right)}^{2}$ = 199.76, *p* < 0.001; this was also true in distant relationships, $\chi_{\left(1\right)}^{2}$ = 112.05, *p* < 0.001. Odds ratios indicated that the odds of young senders in close relationships being perceived as sarcastic were 37.63 times higher than the odds of old senders in close relationships. Compared to old senders in distant relationships, young senders in distant relationships were 14.12 higher in being perceived as sarcastic. Thus, the analysis reveals that in both close and distant sender–receiver relationships, young senders are more likely to be perceived as sarcastic than old senders when delivering emoji-based ambiguous statements.

For older participants, the main effect of literality was significant for old senders, $\chi_{\left(1\right)}^{2}$ = 347.97, *p* < 0.001. The results revealed that older participants perceived emoji-based ambiguous statements 6.04 times non-sarcastically than sarcastically. Relationship was significantly associated with literality, $\chi_{\left(1\right)}^{2}$ = 73.21, *p* < 0.001. The odds of senders in close relationships being perceived as sarcastic were 0.12 as those in distant relationships. Therefore, the analysis indicates that older participants rely on the sender–receiver relationship to determine the literality of emoji-based ambiguous statements.

### The Valence of the Smiling Emoji

**Figure 3:**
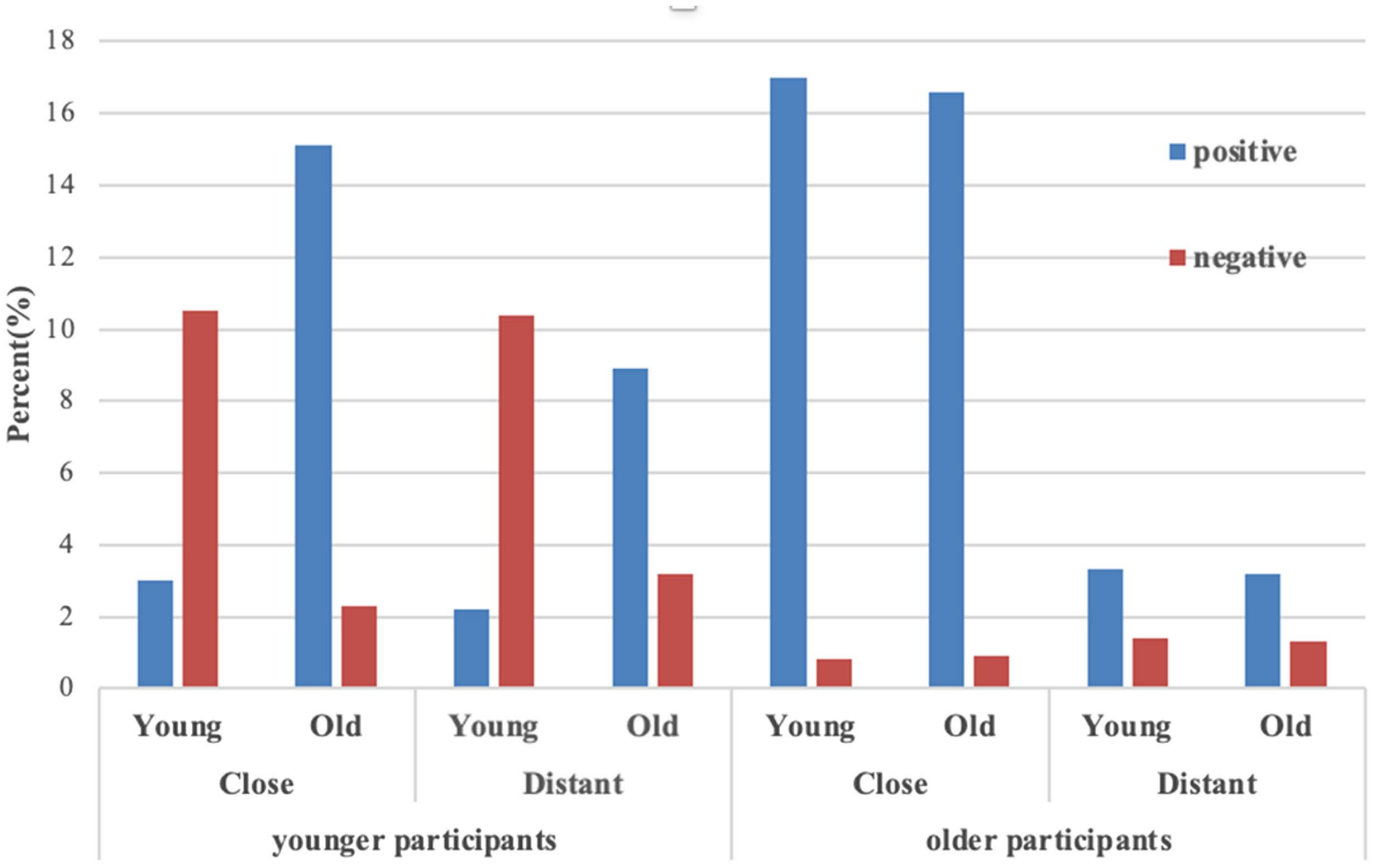
Frequency of the valence of the smiling emoji.

The free response data about the real meaning of the smiling emoji was coded as positive when participants interpreted the emoji the same as its surface meaning (i.e., happiness), but coded as negative when it was interpreted as having an implied negative meaning or simply for politeness or bantering. Therefore, we constructed a 2 (participant age: younger vs. older) ^*^ 2 (relationship: close vs. distant) ^*^ 2 (sender age: young vs. old) ^*^ 2 (the smiling emoji: positive vs. negative) design. We applied log-linear models to analyze the data. The collected data (*N* = 1,376) was more than five times that of the cells (*n* = 16) in multiway tables, which satisfied the criterion of the log-linear model. Figure 3 displays the frequency of each condition.

The results of the goodness-of-fit test showed that the probability (*p* = 0.25) was greater than 0.05, indicating that there was no statistical difference between the unsaturated model and the saturated model with all interactive options. That is, this model fully reflected the relationship between the four variables. The probability value of the likelihood ratio chi-square test showed that the fourth-order and above effects were non-significant ($\chi_{\left(1\right)}^{2}$ = 1.01, *p* = 0.31). Therefore, we broke down the 4-way interaction and performed a chi-square test on relationship, sender age, and literality separately for younger and older participants. For younger participants, there was no significant association among sender age, relationship, and the smiling emoji ($\chi_{\left(1\right)}^{2}$ = 2.34, *p* = 0.13); nor was it significant in older participants ($\chi_{\left(1\right)}^{2}$ = 0.05, *p* = 0.83).

Log-linear results revealed that the third-order and above ($\chi_{\left(5\right)}^{2}$ = 142.65, *p* < 0.001) was significant. There was a significant association among participant age, relationship, and the smiling emoji, $\chi_{\left(1\right)}^{2}$ = 19.81, *p* < 0.001. A significant association was found among participant age, sender age, and the smiling emoji, $\chi_{\left(1\right)}^{2}$ = 66.35, *p* < 0.001. We split the file by participant age variable. Separate chi-square tests on relationship and the smiling emoji were performed for younger and older participants. There was a significant association between relationship and the smiling emoji in younger participants, $\chi_{\left(1\right)}^{2}$ = 10.49, *p* = 0.001; and this was also true for older participants, $\chi_{\left(1\right)}^{2}$ = 54.24, *p* < 0.001. Odds ratios indicated that for younger participants, the odds of perceiving the smiling emoji as positive in close relationships were 1.74 times higher than that in distant relationships, while for older participants, the odds of perceiving the smiling emoji as positive in close relationships were 8.26 times higher than that in distant relationships.

Separate chi-square tests on sender age and the smiling emoji were performed for younger and older participants. There was a significant association between sender age and the smiling emoji in younger participants, $\chi_{\left(1\right)}^{2}$ = 308.08, *p* < 0.001; however, this was not true in older participants, $\chi_{\left(1\right)}^{2}$ = 0.03, *p* = 0.87. Odds ratios showed that for younger participants, the odds of young senders’ conveying a negative meaning were 16.76 times higher than the odds of conveying a positive meaning, and the odds of old senders’ conveying a positive meaning were 17.84 times higher than the odds of conveying a negative meaning. However, for older participants, there was no interaction between sender age and the smiling emoji. This indicated that older participants had a high tendency to perceive the smiling emoji as positive regardless of sender age.

### Rating of Friendliness

ANOVA results demonstrated that the three-way interaction among participant age, relationship, and sender age was not significant, *F*(1, 1,368) = 2.23, *p* = 0.14, $\eta_{p}^{2}$ = 0.002. There was no significant interaction between sender age and relationship, *F*(1, 1,368) = 0.32, *p* = 0.57. However, there was a significant association between participant age and relationship, *F*(1, 1,368) = 17.35, *p* < 0.001, $\eta_{p}^{2}$ = 0.01. A significant association was also found between participant age and sender age, *F*(1, 1,368) = 91.42, *p* < 0.001, $\eta_{p}^{2}$ = 0.06. Then, the effect of sender age and relationship on the judgment of sender attitude were analyzed separately for younger and older participants. Table 5 displays the mean score and standard deviation of each factor measured by both younger and older participants.

**Table 5:** The mean score and standard deviation of friendliness in Experiment 2.

*Participants*	*Relationship*	*Sender age*	
		Young senders	Old senders
Younger	Close	*M* = 4.19, SD = 1.75	*M* = 5.77, SD = 1.19
	Distant	*M* = 3.06, SD = 1.66	*M* = 5.01, SD = 1.66
Older	Close	*M* = 6.00, SD = 1.42	*M* = 6.09, SD = 1.18
	Distant	*M* = 4.37, SD = 2.01	*M* = 4.29, SD = 1.81

The results of younger participants’ interpretation showed that there was a significant main effect in relationship, *F*(1,764) = 74.05, *p* < 0.001, $\eta_{p}^{2}$ = 0.09, revealing senders in close relationships were rated friendlier than senders in distant relationships (MD = 0.95, *p* < 0.001). For older participants, there was a significant effect in relationship*, F*(1,764) = 139.82, *p* < 0.001, $\eta_{p}^{2}$ = 0.19, indicating that senders in close relationships were rated friendlier than senders in distant relationships (MD = 1.71, *p* < 0.001).

For younger participants, sender age exerted significant effects on the judgment of friendliness, *F*(1,764) = 225, *p* < 0.001, $\eta_{p}^{2}$ = 0.25. This indicated that young senders were more likely to be perceived as less friendly than old senders (MD = − 1.76, *p* < 0.001). In contrast, for older participants, sender age was not significantly associated with the friendliness of the statements, *F*(1,764) = 0.002, *p* = 0.97.

In summary, the findings of this experiment suggested that sender age and sender–receiver relationship have interaction effects on younger adults’ perception of emoji-based ambiguous statements. The perceived sarcasm of the statement was negatively related to the emoji and the friendliness ratings. Young senders were more likely to be perceived as sarcastic; they were rated less friendly, and their interpretation of the smiling emoji was more negative than old senders. Old senders in distant relationships tended to be more sarcastic, less friendly, and their understanding of the emoji was less positive than those in close relationships. In contrast, older adults’ perception was only affected by sender–receiver relationship. Senders in distant relationships were more likely to be perceived sarcastically and less friendly, and their perception of the emoji was less positive than those in close relationships. This study has added to note that the understanding of the smiling emoji might be negatively associated with the sarcasm of the statements, and positively related to the perceived friendliness.

## General Discussion

The present study investigated whether individuals’ interpretation of emoji-based ambiguous statements would be altered by senders of different ages and relationships and whether the interaction between sender age and sender–receiver relationship changed because of the varying ages of the participants.

The findings of the two experiments consistently demonstrate that sender age and sender–receiver relationship exerted interaction effects on younger participants’ interpretation of emoji-based ambiguous statements. Our investigation of the perception of sender attitude in the context demonstrates that, among younger participants, sender age and sender–receiver relationship influence the friendliness ratings of the statement. Old senders and senders in close relationships tend to express friendlier attitudes using emoji-based ambiguous statements.

There are differences in older adults’ perceptions in the two experiments. The results of Experiment 1 show that sender age and sender–receiver relationship have null effects on older adults. In contrast, when the two factors and the emoji were salient in the context of Experiment 2, sender age showed little influence on older participants’ interpretation, while the sender–receiver relationship affected their judgments. This result reveals that, like younger participants, older participants believe that senders in distant relationships are more likely to express sarcastic meaning than senders in close relationships when delivering ambiguous statements accompanied by a smiling emoji. For older adults’ perception of senders’ attitude, the results of Experiment 1 show that neither sender age nor sender–receiver relationship has any effect on the perception of the friendliness of the statement. In Experiment 2, in contrast, sender age is a less efficient cue for the identification of sender attitude, whereas the sender–receiver relationship plays an important role. This indicates that senders in close relationships are more likely to be interpreted as conveying a friendlier attitude when delivering ambiguous statements accompanied by a smiling emoji.

In summary, when the factors in the context are sufficiently salient, there are clear distinctions in the interpretations of emoji-based ambiguous statements between younger and older participants. Younger adults would change their interpretations of emoji-based ambiguous statements based on sender age and sender–receiver relationship. However, sender age shows limited influence on older participants’ interpretation, but sender–receiver relationship would have some effect on their interpretation.

Based on the free response question, this disparity might have arisen from participants’ diverse interpretations of the smiling emoji. Our results show that the smiling emoji is a vital factor that could affect the literality of the statements. Among younger participants, young senders are more likely to employ the implicit meaning of the smiling emoji (i.e., the negative meaning), so they have a higher tendency to express a sarcasm through emoji-based ambiguous statements. This was in line with Zhou et al.’s (2017) finding that young adults assigned sarcasm or speechlessness to the smiling emoji, whereas older adults tended to take the emoji as a “genuine smile” that indicated happiness and friendliness. As has been suggested, younger adults’ use of the smiling emoji might not be commonly understood by older adults over 30 years old. Therefore, younger participants seldom bother to send older people the smiling emoji to express sarcasm. Likewise, senders in distant relationships are inclined to use the smiling emoji negatively, so their use of emoji-based ambiguous statements are more likely to be interpreted sarcastically. Similarly, older participants agreed that senders in distant relationships have a higher tendency to express negative meanings when using the smiling emoji. Thus, when they used emoji-based ambiguous statements, their statements were more likely to be interpreted sarcastically.

Another possible explanation might be the notable Confucian teaching of respecting elders, especially for young Chinese adults (Sung, 2000). The nexus of this tradition is deference and reverence toward elderly people. According to The Teachings of Filial Piety (1989), young people show upgraded respect with the increase of target age. Many studies have demonstrated that there were potential links between age stereotypes, filial piety, and younger–older adult communication (e.g., Hummert et al., 2004). Young adults’ perception of age cues has promoted both positive and negative stereotypes of elderly people (Harwood et al., 2000). The personal vitality (e.g., health and activity) of the older people decreases from early adulthood, but the traits of benevolence, such as kindness, wisdom, and generosity, increase with the ascent of age (McCann et al., 2005). However, sarcasm is not set for simply providing information but for conveying the sender’s intention and attitude. Sarcastic statements with positive literal meanings (sarcastic criticism) convey implicit criticism or earlier negative attitudes (Wilson and Sperber, 2012; Dynel, 2013). Therefore, because of the negative nature of sarcasm, older people are less likely to be perceived as sarcastic when delivering ambiguous statements accompanied by a smiling emoji.

Older adults, according to the SST, are supposed to hold a more positive attitude toward life and prioritize more positive emotions than younger adults (Carstensen et al., 2000). People become aware of future mortality in their early adulthood (Carstensen, 2006). Their stable emotions in daily communication result in a lower tendency to utilize irony or sarcasm to express their intentions; instead, they go directly to the goals. This age-related positivity bias in interpreting sarcasm indicates that older adults have a preference for positive interpretation of all potentially ambiguous utterances (Reed et al., 2014). Older people are also more inclined to associate emoticons/emoji with greater positive emotions compared to younger adults (Hsiao and Hsieh, 2014). The smiling emoji is primarily designed as an ordinary smile, so that older adults are inclined to go directly to the surface and have a positive meaning of the genuine smile. Hence, it appears that old adults do not show any specific loving toward the young. Instead, they present their positivity bias toward all things and people in their lives.

The sender–receiver relationship is a factor commonly considered by both younger and older participants. Previous evidence has shown that the close sender–receiver relationship is not significantly associated with the perception of sarcasm (Kreuz et al., 1999; Pexman and Zvaigzne, 2004). The null effect of a close relationship on irony ratings has been explained by Kreuz et al.’s (1999) idea that irony is not a gradient but an all-or-none type of phenomenon. In this study, we employed a sarcastic/non-sarcastic dichotomy to evaluate sender intention and found inconsistent results with Kreuz et al.’s (1999). Our findings demonstrate that both younger and older participants believe that senders in distant relationships are more likely to deliver sarcastic statements to receivers. This might be because of their belief that individuals in distant relationships have a higher tendency to employ the smiling emoji as an indicator of sarcasm than senders in close relationships. It has been suggested that the relationship between people is a prominent determinant of trust (Binzel and Fehr, 2013). People appear to have greater trust in friends, so that socially close persons are perceived to be friendlier in attitude and are less likely to express critical and negative emotions through the emoji. Additionally, according to previous literature, a close relationship is also a more potent factor for ironic compliments than for ironic criticisms (Pexman and Zvaigzne, 2004; Pexman et al., 2010). Therefore, closeness has a limited effect on the superficially positive statements employed in the present study. Moreover, relationship might be a variable component of affective valence that interacts with other variables to construct the background relationship context.

Relationship is not an isolated factor affecting sarcasm interpretation. However, it might interact with the other factors and jointly compose the background relationship context. Relationships are claimed to be an integral part of liking (Slugoski and Turnbull, 1988). That is, those who liked each other tended to be more intimate in their relationships than the disliked people. Intimate relationships would not facilitate the non-literal interpretation of superficially positive statements, but when directed at disliked receivers, these statements are more likely to be interpreted ironically. The sender–receiver relationship showed null effects on the interpretation of ironic criticism; however, when considering the directness of irony, there were interaction effects between relationship and directness of ironic remarks (Pexman et al., 2010). In close relationships, irony was perceived more strongly for direct ironic remarks, whereas in distant relationships, irony was perceived to be stronger for indirect ironic remarks. Our study adds to the existing literature that, apart from the sender–receiver relationship, the ages of both the sender and the recipient (i.e., participants) influence the sarcastic interpretation of emoji-based ambiguous statements.

## Conclusion

This study reflects the complexity of sarcasm interpretation and takes into consideration the relationship between the characters and the ages of both the sender and the participants. Our findings reveal that younger recipients are more sensitive to sender age compared with older participants. Both groups show that senders in distant relationships are more likely to use sarcasm than individuals in close relationships when sending ambiguous statements accompanied by a smiling emoji. This is in line with the Confucian teaching of “respecting the old.” Younger adults interpreted young senders’ emoji-based ambiguous statements sarcastically, and they regarded old senders as friendlier in attitude when delivering emoji-based ambiguous statements. Only in distant relationships are old senders perceived to express sarcastic intentions. Older participants, however, show no biased love toward the young as the Confucian doctrine instructs. Instead, they regard both the young and the old alike, claiming that senders in distant relationships would slightly outperform those in close relationships in expressing sarcastic intentions.

The findings provide evidence for the constraint satisfaction model and incorporate both age and relationship as two social factors that are beneficial to sarcasm interpretation. We also highlighted the interactions between these two factors in helping to interpret emoji-based ambiguous statements. In addition, this study is the first to delegate powers to participants themselves in making judgments of sender age and sender–receiver relationship based on their personal experiences, allowing them to set up their exclusive background relationship context. This, in turn, would avoid disagreements between researchers and participants in deciding sender age and sender–receiver relationship.

There are some limitations of this study that should be noted. First, future research should enlarge the sample size and cover a more diverse population to strengthen the representativeness of the samples. Second, the distribution of age is another possible limitation. The present study classified 40 to 59 years old as older groups which might have a relatively lower limit. Further research is needed to recruit older participants over 60 years. Another limitation of the study is that the three factors—age, relationship, and the smiling emoji—were designed as dichotomies, namely, “young/old age,” “close/distant relationship,” and “positive/negative emoji.” It leaves out the continuum part in between the two extreme states. Future research should include other graded conditions into the test, for instance, including “neutral” into the valence of emoji, so that an overall view can be achieved. In addition, people in Chinese culture emphasize social relationships and produce communication patterns based on Confucian doctrines. However, other cultures that do not follow Confucian doctrines but rather emphasize on individualism might produce very different interpersonal relationships and communication patterns. Thus, a cross-cultural study about the influence of these diverse communication patterns in sarcasm interpretation is needed in the future.

## Data Availability Statement

The datasets presented in this study can be found in online repositories. The names of the repository/repositories and accession number(s) can be found at: https://osf.io/y3apv/?view_only=7bbdfed281754975b3bddad9241629df.

## Ethics Statement

The studies involving human participants were reviewed and approved by College of Foreign Languages and Cultures, Xiamen University, China. The patients/participants provided their written informed consent to participate in this study.

## Author Contributions

The author confirms being the sole contributor of this work and has approved it for publication.

## Conflict of Interest

The author declares that the research was conducted in the absence of any commercial or financial relationships that could be construed as a potential conflict of interest.

## Publisher’s Note

All claims expressed in this article are solely those of the authors and do not necessarily represent those of their affiliated organizations, or those of the publisher, the editors and the reviewers. Any product that may be evaluated in this article, or claim that may be made by its manufacturer, is not guaranteed or endorsed by the publisher.

## Footnotes

^1^*Orders of relationship*: in the parlance of Confucianism, regardless of who one interacts with, one must follow the rules of ordering — the youngster must show respect to the elderly. Besides, different orders of interacting are based upon whether the relationship is close or distant.

^2^The WeChat app is the most popular instant messaging product in China, and it has become an integral component of Chinese people’s personal life. As at August 2021, there were over 1.25 billion monthly active users.

^3^*Speechlessness* means the state of not bothering to say anything when one cannot understand other people’s extreme words or behavior.
